# An estimate assay for low-level exposure to ionizing radiation based on mass spectrometry quantification of γ-H2AX in human peripheral blood lymphocytes

**DOI:** 10.3389/fpubh.2022.1031743

**Published:** 2022-10-28

**Authors:** Hongling Zhao, Minmin Qu, Yuchen Li, Ke Wen, Hua Xu, Man Song, Dafei Xie, Xingkun Ao, Yihao Gong, Li Sui, Hua Guan, Pingkun Zhou, Jianwei Xie

**Affiliations:** ^1^Beijing Key Laboratory for Radiobiology, Beijing Institute of Radiation Medicine, Beijing, China; ^2^State Key Laboratory of Toxicology and Medical Countermeasures and Laboratory of Toxicant Analysis, Institute of Pharmacology and Toxicology, Beijing, China; ^3^Department of Nuclear Physics, China Institute of Atomic Energy, Beijing, China

**Keywords:** low-dose exposure, ionizing radiation, γ-H2AX, human peripheral blood lymphocytes, mass spectrometry

## Abstract

Exposure to environmental ionizing radiation (IR) is ubiquitous, and large-dose exposure to IR is known to cause DNA damage and genotoxicity which is associated with an increased risk of cancer. Whether such detrimental effects are caused by exposure to low-dose IR is still debated. Therefore, rapid and early estimation of absorbed doses of IR in individuals, especially at low levels, using radiation response markers is a pivotal step for early triage during radiological incidents to provide adequate and timely clinical interventions. However, there is currently a crucial shortage of methods capable of determining the extent of low-dose IR exposure to human beings. The phosphorylation of histone H2AX on serine 139 (designated γ-H2AX), a classic biological dosimeter, can be used to evaluate the DNA damage response. We have developed an estimation assay for low-level exposure to IR based on the mass spectrometry quantification of γ-H2AX in blood. Human peripheral blood lymphocytes sensitive to low-dose IR, maintaining low temperature (4°C) and adding enzyme inhibitor are proven to be key steps, possibly insuring that a stable and marked γ-H2AX signal in blood cells exposed to low-dose IR could be detected. For the first time, DNA damage at low dose exposures to IR as low as 0.01 Gy were observed using the sensitive variation of γ-H2AX with high throughput mass spectrometry quantification in human peripheral blood, which is more accurate than the previously reported methods by virtue of isotope-dilution mass spectrometry, and can observe the time effect of DNA damage. These *in vitro* cellular dynamic monitoring experiments show that DNA damage occurred rapidly and then was repaired slowly over the passage of post-irradiation time even after exposure to very low IR doses. This assay was also used to assess different radiation exposures at the *in vitro* cellular level. These results demonstrate the potential utility of this assay in radiation biodosimetry and environmental risk assessment.

## Introduction

Since the discovery of X-rays by German physicist Roentgen (1), industries that utilize radiation and radioactive materials such as the medical, agriculture, and nuclear power industries have been expanding (2–4). On the other hand, accidental or occupational exposure to ionizing radiation (IR), which occurs to individuals during nuclear mishaps, to astronauts, and to some medical professionals, can cause side effects (5–7). Radiation therapy, one of the most important therapeutic strategies for treating malignancies, can also injure the normal cells and tissues surrounding tumors (8). Therefore, IR from natural and artificial sources is a double-edged sword in our daily life.

Exposure to moderate-to-high doses of IR is known to induce genotoxic effects that can lead to carcinogenesis (9). However, whether such detrimental effects can be produced after exposure to low-dose IR is still controversial (10). Usually, a low dose of IR is a radiation dose of 100 mSv or less (≤ 100 mGy) (11). Epidemiological and clinical studies show that low-dose IR may induce cancer, cardiovascular diseases and long-term psychological consequences (11). For example, increased risk of leukemia and brain tumors have been reported in pediatric patients following doses of 30–50 mGy, as observed using CT scans (12). There are still questions, however, about the impact of even lower doses of exposure for which classical epidemiological studies are limited. In addition, when the health risks associated with exposure to low-dose IR were estimated in previous studies, there were many uncertainties (11, 13). These uncertainties significantly affect almost every facet of society, especially medical care, energy production, occupational health and safety, manufacturing and industry, and all these factors emphasize the importance of low-dose IR research (10, 13, 14).

When an individual is exposed to IR, knowledge of the absorbed dose is essential for early triage during radiological incidents to provide optimum, potentially life-saving procedures. Toward this goal, biological dosimetry methods such as the dicentric assay, micronucleus assay, fluorescence *in situ* hybridization translocation assay, and premature chromosome condensation have been established and used in real-life exposure cases over the past several decades (15, 16). Among the available assays, the dicentric assay remains the international gold standard for biodosimetry measurement of recent radiation exposure (17), detecting exposures to 0.1 Gy when up to 1,000 cells are analyzed (18). Other assays have been explored but lack sensitivity in the low-dose range (19–21). In addition, the established methods still have disadvantages in large-scale accidents, with the most pressing issues being the time it takes to culture blood samples and the applicable dose range (22, 23). The culture time for analysis using the premature chromosome condensation fusion method has been reduced to 2 h, but this is technically difficult, expensive, and restricted to high doses (24).

However, the most common IR exposure encountered by humans, both in the environment and occupationally, is protracted or chronic low-dose exposure. Over the years, a number of molecular, metabolomic, lipidomic, and protein markers exhibiting dose responses have been identified (25–29). None have demonstrated an adequate capability for dose reconstruction in low-dose exposures. γ-H2AX, which is formed by the phosphorylation of Ser 139 in histone H2AX, has been identified as a robust biomarker for DNA double-strand breaks (DSBs) (30). The potential of the γ-H2AX assay for triage and/or to measure the absorbed radiation dose for exposed individuals has been reported in several *in vivo* and *in vitro* studies (31–34). Since the role of γ-H2AX was first elucidated ~2 decades ago, an immunocytochemical assay with antibodies recognizing γ-H2AX, which is sensitive to detect even mGy of IR exposure, has emerged as the gold standard for the *in situ* detection of DNA damage, specifically DSBs (30, 35–37). However, the specificity of immunoassays is limited due to poor batch-to-batch reproducibility as well as some cross-reactivity that originates from antibodies, and accurate quantification is still challenging (34).

**Figure 1:**
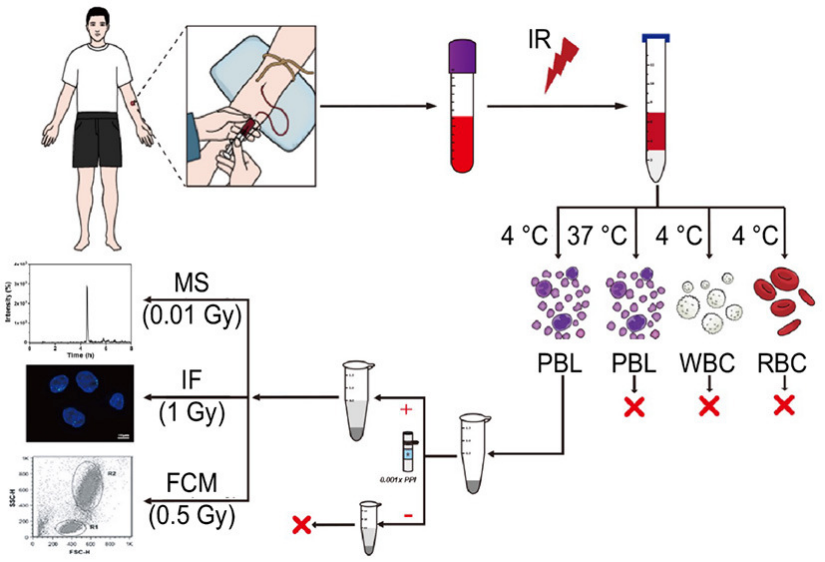
Schematic diagram of an estimation assay for low-dose exposure to IR based on mass spectrometry quantification of γ-H2AX in blood. Blood from healthy donors, which was irradiated *ex vivo*, was used to isolate peripheral blood lymphocyte (PBL), white blood cell (WBC) and red blood cell (RBC) at 4°C or 37°C according to the manufacturer's instructions of the human lymphocyte separation medium and the red blood cell lysate. Subsequently, after nuclear isolation with or without protease inhibitors, histone extraction, trypsin digestion in the solution, and desalting, the peptide sample from the carboxy terminus of H2AX was used for LC–MS/MS analysis.

We have established a sensitive and convenient liquid chromatography-tandem mass spectrometry (LC–MS/MS) method to detect cellular γ-H2AX for use as a tool to rapidly screen the genotoxicity or carcinogenicity of chemicals (38–40). Here, an estimation assay based on the mass spectrometry quantification of γ-H2AX is developed to determine whether DNA damage and its profiles are caused under very low dose exposure to IR (as illustrated in Figure 1). Using human blood cells, optimization of γ-H2AX extraction and other experimental manipulations were performed and the DNA damage effect at low levels of exposure to IR was investigated. This assay promises potential utility in radiation biodosimetry and environmental risk assessment for low-dose IR exposures.

## Materials and methods

### Materials and reagents

Peptides that represented the sequences of trypsin cleavage products from γ-H2AX and H2AX (ASQA*p*SQEY and ASQASQEY), together with the corresponding isotope-labeled peptides containing ^13^C3 as well as ^15^N-labeled N-terminal alanine, i.e., (^13^C3, ^15^N) ASQA*p*SQEY and (^13^C3, ^15^N) ASQASQEY, were provided by Sangon Biotech Co., Ltd. (Shanghai, China). Trypsin of sequencing grade was provided by Promega Biotech Co., Ltd. (Beijing, China). Mouse monoclonal antibodies against γ-H2AX were purchased from Millipore (Shanghai, China) and diluted 1:200 for use. Alexa Fluor 488-labeled goat anti-mouse IgG (H + L) secondary antibodies were obtained from Sigma–Aldrich Inc. (MO, USA) and diluted 1:5,000. The C18 Empore disk was provided by 3 M (Beijing, China). Protease inhibitor cocktails and phosphatase inhibitors were purchased from Roche (Shanghai, China).

Dulbecco's modified Eagle's medium (DMEM), Roswell Park Memorial Institute Medium 1,640 (RPMI 1,640), penicillin/streptomycin, and fetal bovine serum (FBS) were purchased from Life Technologies (Paisley, UK). Red blood cell lysate and human lymphocyte separation medium were purchased from Tianjin Hao Yang Biological Manufacture Co., Ltd. (Tianjin, China). A nuclear extraction kit was provided by Solar Bio S&T Co., Ltd. (Beijing, China). Formic acid (FA) was obtained from Sigma–Aldrich Inc. (MO, USA). Other compounds or reagents were purchased from Sinopharm Chemical Reagents Co., Ltd. (Beijing, China).

### Cell culture and radiation exposure

Human lymphoblast cells (AHH1) and human bronchial epithelial cells (16HBE) were grown in RPMI 1,640 and DMEM, respectively. Both media were supplemented with 10% (v/v) FBS, 100 U mL^−1^ penicillin and 100 μg mL^−1^ streptomycin. Cultures were maintained in a humidified atmosphere with 5% CO_2_ at 37°C, and the medium was refreshed every 2–3 days during subculturing. All experiments were performed with exponentially growing cells, and the passage number was below 25.

Initially, AHH1 and 16HBE cells were irradiated in a low-dose range (0.01, 0.02, 0.05, 0.1 and 0.2 Gy) at a dose rate of 0.0281 Gy/min using ^60^Co γ-ray irradiation (0.24 keV/μm in water) to evaluate low-dose IR-induced γ-H2AX levels and DNA damage. Sham-exposed cells were used as controls. Cells were incubated at room temperature (RT) for various designated post-exposure times (0.5, 1, 2, 4, 8, and 24 h) until further processing for γ-H2AX MS analysis.

For low-linear energy transfer (LET) irradiation in the space radiation environment, a 100 MeV proton cyclotron (0.7371 keV/μm in water) provided by the China Institute of Atomic Energy was used. The dose rate of protons was 0.8 Gy/min. For high-LET irradiation in the space radiation environment, a heavy-ion beam ^12^C^6+^ (30.79 keV/μm in water) developed by the Institute of Modern Physics, Chinese Academy of Science, was used. The dose rate for heavy-ion beam ^12^C^6+^ was 1 Gy/min. AHH1 cells were exposed to varying proton and heavy-ion doses (sham, 0.5, 1, 2, 4, and 6 Gy) to evaluate the response for space radiation. Cells were collected at various designated post-exposure times (1, 2, 4, 8, and 24 h) and then processed for γ-H2AX MS analysis.

### Human peripheral blood sampling and processing

After obtaining written informed consent from volunteers and ethical approval from the Subcommittee on Human Investigation of the Beijing Institute of Radiation Medicine (AF/SC-08/02.146), we collected peripheral blood samples from 41 healthy adults volunteers (16 females and 25 males ranging from 20 to 40 years old) by venipuncture into Vacutainer tubes that contained ethylene diamine tetraacetic acid as an anticoagulant. The subjects had no history of chronic disease, substance abuse, smoking, or toxic chemical exposure. Furthermore, they had not been exposed to radiation and had no history of viral infections during the 6 months preceding the study.

First, healthy human blood samples that were not irradiated were obtained to measure the basal level of γ-H2AX. Blood was drawn from each volunteer and kept at RT for 30 min to 1 h to isolate lymphocytes and white blood cells (WBCs) at 4°C according to the manufacturer's instructions for the human lymphocyte separation medium and the red blood cell lysate. Then, blood samples from the same 20 volunteers, randomly selected from the 41 non-smoking healthy volunteers, were used for all radiation exposure experiments. The blood samples were divided into 2 mL aliquots and then irradiated with 4 Gy at a dose rate of 0.6661 Gy/min and a low-dose range (0.01, 0.02, 0.05, 0.1, and 0.2 Gy) at a dose rate of 0.0281 Gy/min using ^60^Co γ-ray irradiation (0.24 keV/μm in water). Sham-irradiated and irradiated blood samples were treated in the same way. Afterward, the aliquots were recovered at RT for 0.5 h, and the cellular histones of lymphocytes and WBCs were extracted with a nuclear extraction kit, which included the addition of protease inhibitor and phosphatase inhibitor at 4°C, and prepared for subsequent γ-H2AX MS analysis.

### Determination of γ-H2AX based on MS analysis

The determination of γ-H2AX based on the MS technique was performed as previously described (38, 39). Briefly, cells were washed and collected in 1.5-mL tubes after treatment. After nuclear isolation, histone extraction, trypsin digestion in the solution, and desalting, the peptide sample from the carboxy terminus of H2AX was analyzed. LC–MS/MS analysis was conducted using a QTRAP 5,500 (AB Sciex, Framingham, USA) with an ACQUITY UPLC system (Waters Co., Manchester, UK). Chromatographic separation was carried out with an ACQUITY UPLC BEH C18 column (100 mm × 2.1 mm, 1.7 μm). The column temperature was maintained at 40°C. A 10 μL sample aliquot was injected for analysis. Mobile phases A and B were 0.1% FA in distilled H_2_O and acetonitrile, respectively. The elution gradient was initiated with 1% B and linearly increased to 30% B in 8 min at a flow rate of 0.25 mL/min. The eluent composition was maintained for 2 min, after which the system returned to 1% B and was re-equilibrated for 2 min. The eluates in the first 1 min were switched to waste to prevent contaminating the ion source. The electrospray ionization (ESI) source was operated in positive mode using nitrogen as the nebulizing gas. All experiments were performed independently in at least triplicate.

### Immunofluorescence

AHH1 cells were cultured in 25 mm culture bottles and treated with varying γ-ray doses (sham, 1, 2, 4, and 8 Gy) at a dose rate of 0.6661 Gy/min using ^60^Co γ-ray irradiation (0.24 keV/μm in water). The cells were washed three times with precooled phosphate buffered saline (PBS) 1 h after IR was applied and were spotted onto slides with a cytospin. The cells were subsequently permeabilized with PBS that contained 0.5% Triton X-100 at RT for 10 min and were blocked with 10% FBS in PBS at RT for 1 h. Then, the cells were incubated for 1 h with γ-H2AX primary antibody at RT. The cells were subsequently washed three times with PBS and then incubated with Alexa Fluor 488-labeled goat anti-mouse IgG (H+L) secondary antibody. Then, 4′,6-diamidino-2-phenylindole (DAPI) staining was performed. Slides were imaged using a NIKON TI2-E (Nikon, Japan) and CRESTOPTICS X-LIGHT V3 fluorescence microscope system (Crest Optics, Italy).

### Statistical analysis

The results are expressed as the mean ± standard deviation (SD) and were calculated from the quantitative data obtained from three replicate experiments. Statistical analysis was performed using one-way analysis of variance in SPSS version 21.0 software (IBM Corp., Armonk, NY, USA). The significance of the differences between groups was determined using one-way analysis of variance (ANOVA). *P* < 0.05 was considered statistically significant, and n.s. means not significant. Graphs were generated using Origin 8.0. Linear regression analysis and curve fitting were conducted using SAS 9.4 (SAS Inc.), and the respective figures were generated in Origin 8.0.

## Results

### Dose and time response for γ-H2AX after low doses of γ-ray exposure in cells

We previously reported a sensitive and convenient method for detecting cellular γ-H2AX based on stable isotope dilution mass spectrometry (38). This method can dynamically monitor DNA damage and repair processes for genotoxic compounds by the amount change of γ-H2AX and assess the potential carcinogenicity of genotoxic compounds from the European Center for the Validation of Alternative Methods list (39). To investigate whether this method could be used to evaluate low-dose IR-induced γ-H2AX levels and DNA damage, we administered a variety of low doses of IR to two cell lines, AHH1 and 16HBE.

**Figure 2:**
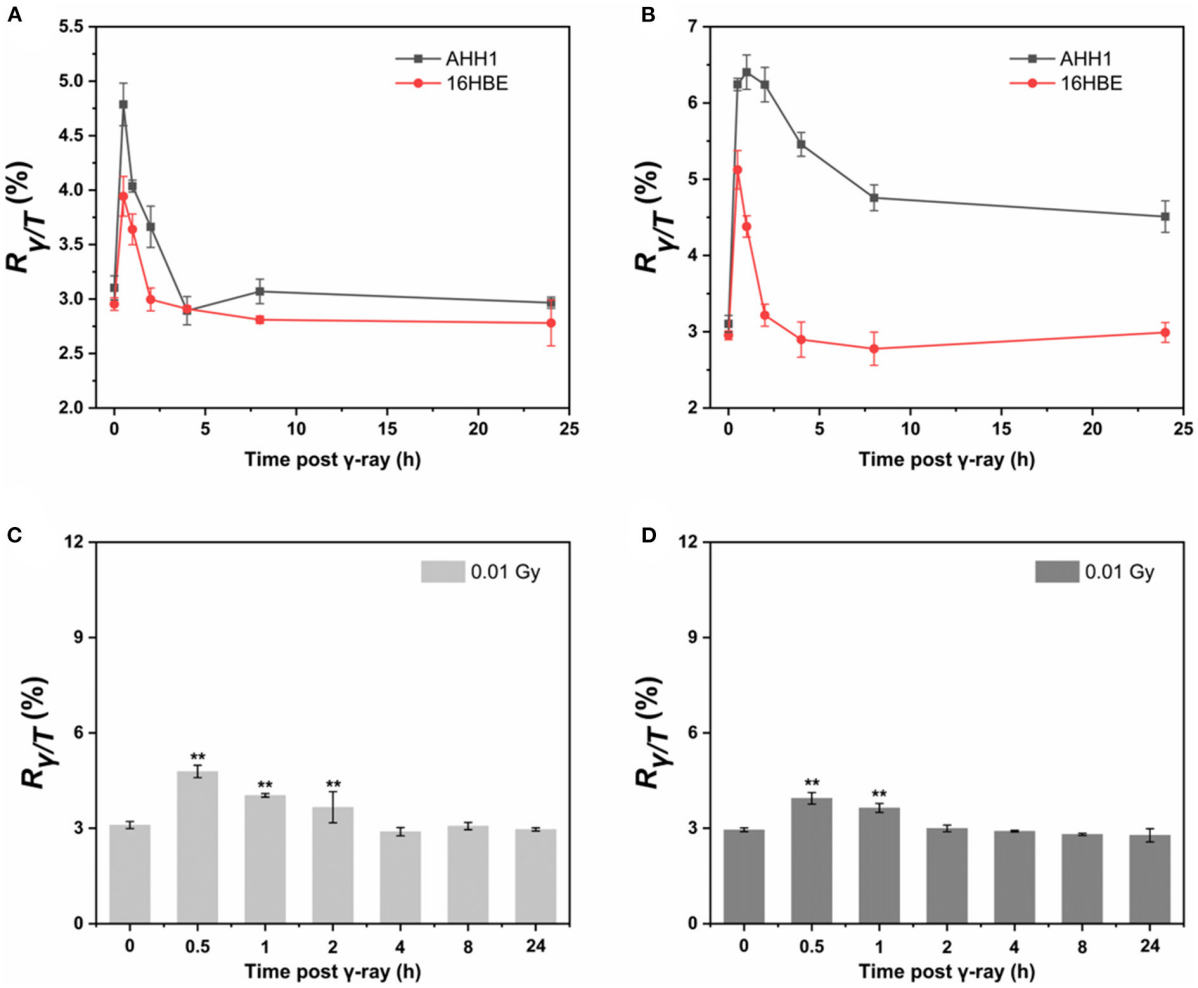
Time courses of γ-H2AX in AHH1 and 16HBE cells treated with **(A)** 0.01 Gy and **(B)** 0.2 Gy of γ-ray irradiation for six time points: 0.5, 1, 2, 4, 8 and 24 h. Time courses of γ-H2AX in **(C)** AHH1 and **(D)** 16HBE cells treated with 0.01 Gy of γ-ray irradiation for six time points: 0.5, 1, 2, 4, 8 and 24 h. The horizontal axis represents the exposure time for γ-ray, and the vertical axis represents the proportion of the number of phosphorylated peptides to the total number of peptides in a cell (*n* ≥ 3, mean ± SD, **p ≤ 0.01).

The results show that the sensitivity of AHH1 and 16HBE cells to radiation is different, as γ-H2AX levels in AHH1 cells increased in a dose-dependent manner after 0.01–0.2 Gy γ-ray exposure, and the levels in 16HBE cells were less affected (Supplementary Figure 1). Since post-irradiation kinetics must be considered when using γ-H2AX in biodosimetry (34), the changes in γ-H2AX for 0.01 and 0.2 Gy γ-ray irradiation were investigated at six time points over a 24 h period in AHH1 and 16HBE cells (Figure 2). At 0.5 h post-irradiation, the proportion of γ-H2AX in a cell sharply increased to a peak and then gradually decreased. An obvious difference between the two types of cells is that the γ-H2AX level in AHH1 cells increased to a greater extent than in 16HBE cells at 0.5 h post-irradiation, further suggesting that the γ-H2AX levels in AHH1 cells are more sensitive to low levels of IR exposure (41).

### Utility of γ-H2AX for evaluation of the response after low-LET and high-LET IR in AHH1 cells

**Figure 3:**
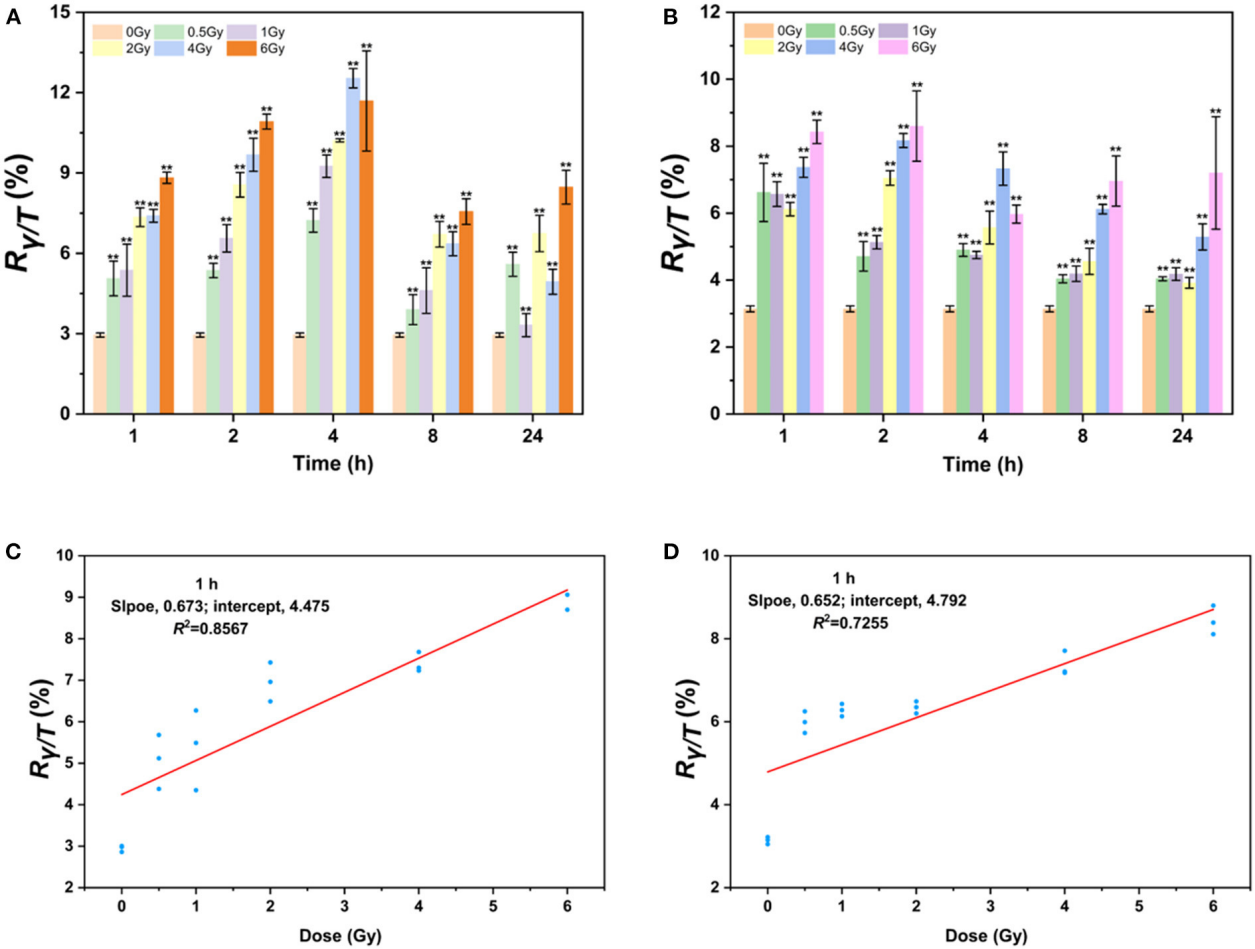
γ-H2AX response after low-LET proton and high-LET heavy ion exposure in AHH1 cells. **(A)** γ-H2AX change in 0.5–6 Gy proton-irradiated AHH1 cells at various time points, **(B)** γ-H2AX change in 0.5–6 Gy heavy ion-irradiated AHH1 cells at various time points, **(C)** linear regression plot of γ-H2AX levels in proton-irradiated AHH1 cells vs. doses at 1 h post-exposure, and **(D)** linear regression plot of γ-H2AX levels in heavy ion-irradiated AHH1 cells vs. doses at 1 h post-exposure (*n* ≥ 3, mean ± SD, **p ≤ 0.01).

Since AHH1 cells are more suitable for γ-H2AX research, the γ-H2AX levels in AHH1 cells after proton and heavy-ion exposure were further profiled. As seen in Figure 3, the γ-H2AX levels increased significantly compared to those of sham-exposed cells when AHH1 cells were exposed to 0.5–6 Gy proton and heavy-ion irradiation at various time points, but these presented different respective trends. The γ-H2AX levels under these two types of ion irradiation all showed a definite dose-dependent relationship, but a poor relationship appeared for heavy ions because of severe cell deterioration and even death. Furthermore, we observed a distinct difference in the peak time of the time-effect relationship. The peak time for heavy ions was ~1 or 2 h (Figure 3B), while there was a time delay for 100 MeV protons, with peak time occurring at 4 h (Figure 3A). Significant differences in the peak time for the two types of irradiations further suggests that damage to cells from heavy ions was more hazardous than that from protons (42).

To further evaluate the usefulness of γ-H2AX as a potential biodosimeter for space radiation, we constructed two dose-effect calibration curves. The equations for trend lines and *R*-squared values are *y* = 0.673x + 4.475/*R*^2^ = 0.8567 and *y* = 0.652x + 4.792/*R*^2^ = 0.7255 in the dose range of 0–6 Gy irradiation at 1 h post-exposure, respectively, where y is the ratio of γ-H2AX to total H2AX (R_γ−*H*2*AX*/*TotalH*2*AX*_, briefly, *R*_γ/*T*_) and x is the dose (Figures 3C,D). Compared to heavy ions, the correlation coefficient of the fitted curve for protons is better, which may be attributed to the less obvious dose-effect relationship in the latter stage of cells irradiated by heavy ions.

### Correlation analysis of immunofluorescence and LC-MS/MS for γ-H2AX

**Figure 4:**
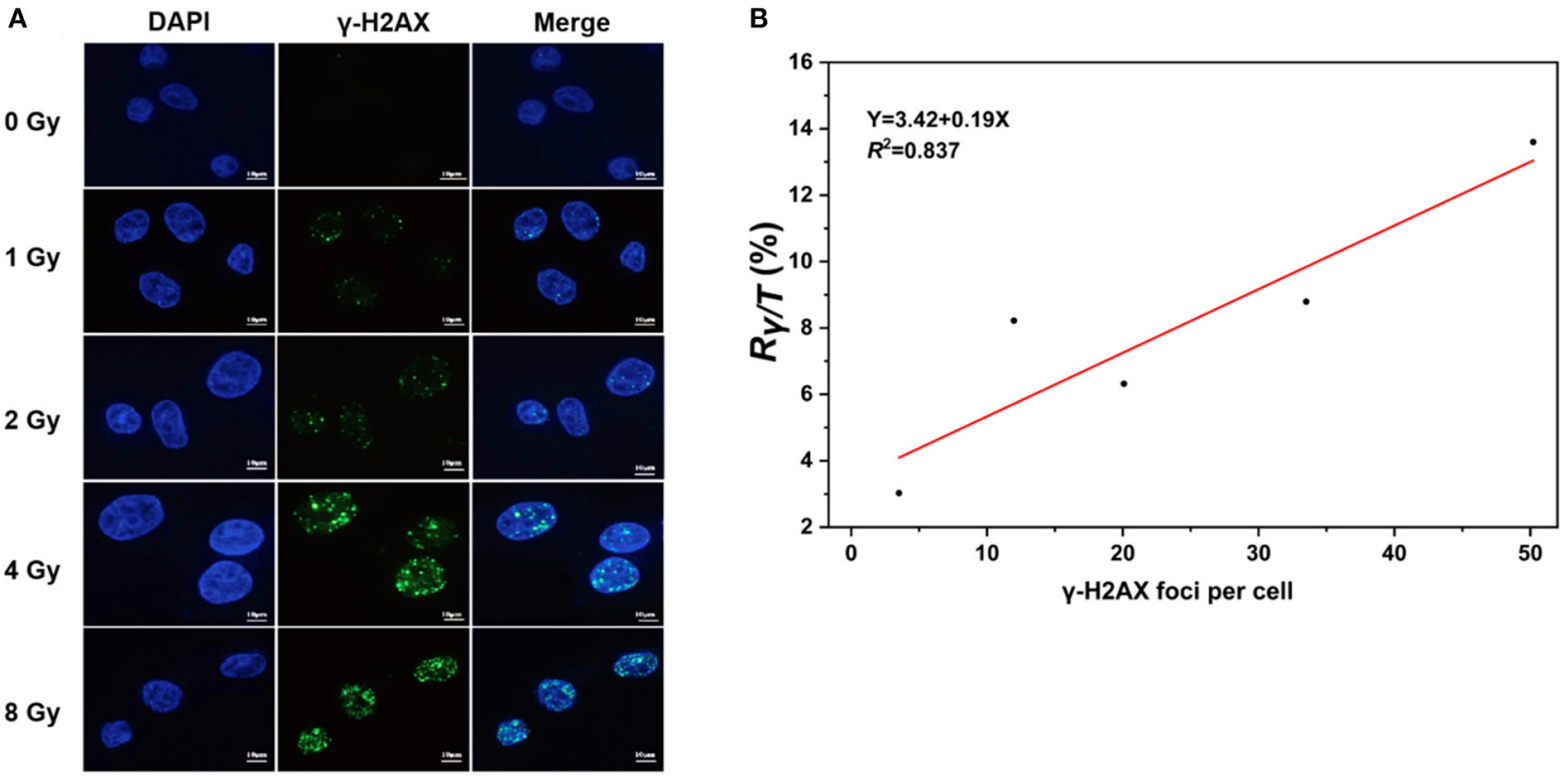
Correlation analysis of immunofluorescence and LC–MS/MS methods for the measurement of γ-H2AX, **(A)** γ-H2AX foci of immunofluorescent staining in AHH1 cells exposed to γ-ray in the dose range of 0–8 Gy at 0.5 h post-irradiation, and **(B)** the correlation analysis of immunofluorescence and LC–MS/MS in AHH1 cells exposed to γ-ray in the dose range of 0–8 Gy at 0.5 h post-irradiation.

The levels of γ-H2AX in cells exposed to IR are commonly measured with immunoassays, including immunofluorescence staining, western blot techniques, flow cytometry, and enzyme-linked immunosorbent assays (34). To further verify whether the levels of γ-H2AX are accurately and quantitatively evaluated with LC–MS/MS assays, we determined the γ-H2AX foci number in AHH1 cells exposed to γ-ray using immunofluorescence and performed correlation analysis of immunofluorescence and LC–MS/MS assays (Figure 4A). As expected, the number of γ-H2AX foci increased with the radiation dose, with a correlation coefficient of 0.837 between immunofluorescence and LC–MS/MS for γ-H2AX analysis (Figure 4B), suggesting a strong correlation between the two analysis assays.

### Extraction conditions for γ-H2AX detection in human peripheral blood lymphocytes

To further verify the practicability of LC-MS/MS to detect γ-H2AX levels in blood cells, we used human peripheral blood samples. However, it was found that the γ-H2AX signal could not be detected in either WBCs or lymphocytes using previously published protocols (38). Therefore, the choice of blood cells sensitive to IR exposure, cell lysis medium, and condition optimization were investigated using human blood exposed to IR at low levels.

First, a series of extraction conditions for the influence of the γ-H2AX signal were performed and optimized, which included different concentration gradients and combination ratios for protease and phosphatase inhibitors (Supplementary Table 1), volumes of blood and temperatures (from 4 to 37°C). The results show that the addition of 1 × protease inhibitor and at least 0.001 × phosphatase inhibitor at 4°C gave an optimal γ-H2AX signal in lymphocytes (Supplementary Table 1).

**Figure 5:**
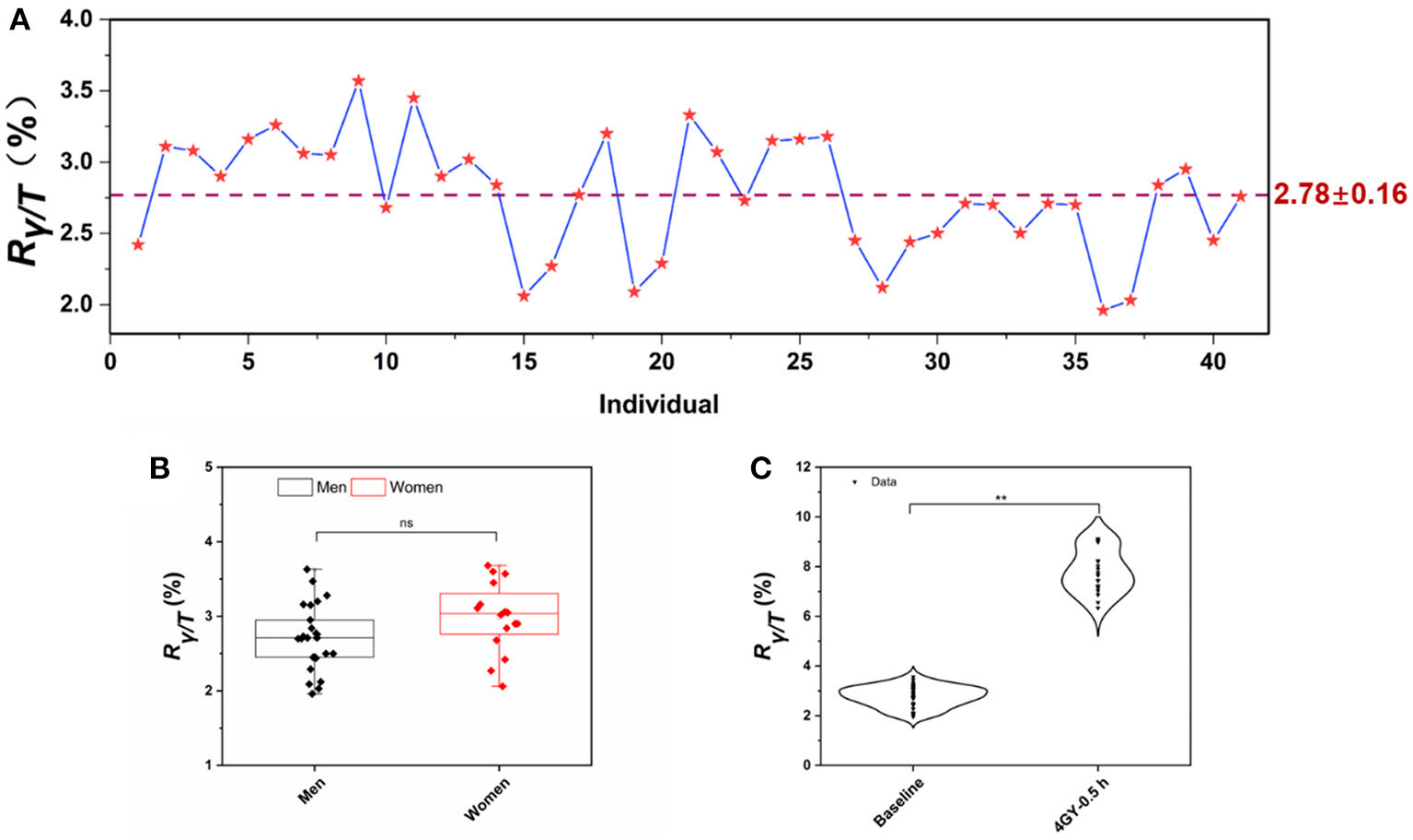
γ-H2AX signal in healthy human donors. **(A)** The background levels of *R*_γ/*T*_ in peripheral blood lymphocytes from 41 healthy human donors, **(B)** dot-whisker plot of γ-H2AX levels between gender groups, and **(C)** comparison of γ-H2AX levels in the lymphocytes of peripheral blood exposed to γ-ray in the dose of 4 Gy at 0.5 h post-irradiation with those of sham-exposed blood. (*n* ≥ 3, mean ± SD, ^ns^*p* > 0.05, **p ≤ 0.01).

Unsorted WBCs exhibited a weak γ-H2AX response compared to that of lymphocytes, reflecting the fact that lymphocytes are a sensitive and well-established blood cell for the γ-H2AX assay in whole blood samples, which is consistent with the fact that lymphocytes are sensitive to IR exposure (41). Furthermore, we drew different volumes of blood to test the γ-H2AX response, to evaluate the sensitivity or limit of quantitation (LOQ) of the assay. As shown in Supplementary Table 2, a 2 mL blood volume was required to accomplish a reliable detection sensitivity, according to the mean value of the background of sham-exposed cells as the LOQ. The *R*_γ/*T*_ for peripheral blood lymphocytes from 41 healthy human donors was determined for the purpose of evaluating and confirming the background levels of γ-H2AX in fresh whole blood. The mean value of *R*_γ/*T*_ in normal human donors was 2.78 ± 0.16 (Figure 5A). In addition, our data also indicate that there is no significant difference in the γ-H2AX signal between gender groups (Figure 5B). Next, fresh whole blood from 20 healthy individuals was irradiated with 4 Gy of γ-ray. Lymphocytes were prepared, and the γ-H2AX signal was measured (Figure 5C). γ-H2AX levels increased 2.8-fold at 0.5 h post-irradiation compared to those of the controls.

### γ-H2AX response at low doses exposure of γ-ray in human peripheral blood lymphocytes

**Figure 6:**
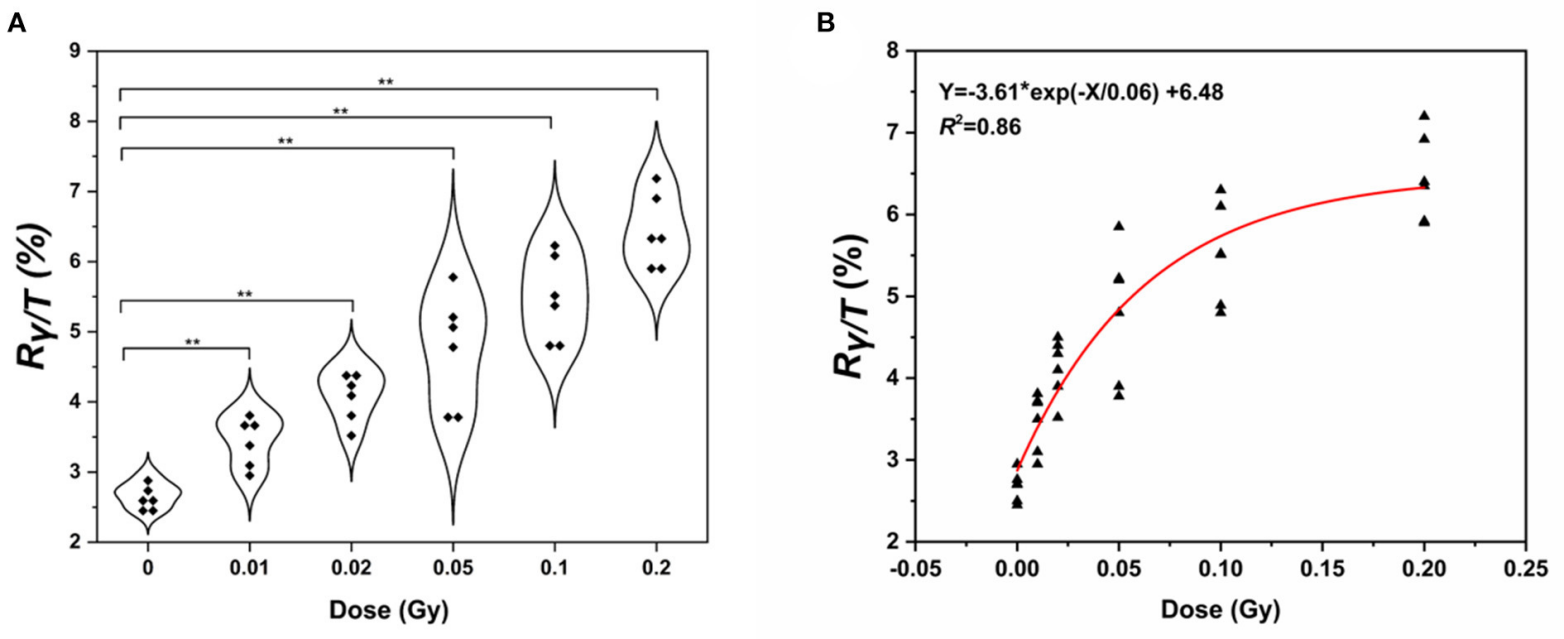
Detection of γ-H2AX levels in human peripheral blood lymphocytes. **(A)** Violin chart and **(B)** fitting curve of γ-H2AX in the lymphocytes of peripheral blood exposed to low doses of γ- rays of 0.01–0.2 Gy γ-ray (*n* ≥ 3, mean ± SD; **p ≤ 0.01, *represents multiplication).

Next, we wanted to determine whether the assay could be used to measure the γ-H2AX response after low-dose exposure to γ-ray. Fresh whole blood from six healthy subjects was irradiated with γ-ray at doses of 0, 0.01, 0.02, 0.05, 0.1, and 0.2 Gy, respectively. Lymphocytes were separated, and the γ-H2AX response was analyzed at 0.5 h post-irradiation (Figure 6A). We observed a significant increase in γ-H2AX levels at 0.5 h compared to that of the control. The γ-H2AX signal could be detected at exposures as low as 0.01 Gy. On this basis, we plotted a fitting curve for the low-dose radiation in human peripheral blood samples (Figure 6B). The correlation coefficient was 0.86, indicating a good degree of fitting.

## Discussion

It is well-known that exposure to large-dose IR increases the risk of cancer and, at higher doses, diseases such as cardiovascular diseases and cataracts (16). However, there are important unanswered questions that need addressing to increase our understanding of the impact of low dose (11). The reason for this consequence is partly due to a crucial shortage of estimate methods for IR exposure to human beings at low-dose levels. We recently developed a sensitive and convenient method to detect cellular γ-H2AX for use as a tool to rapidly screen the genotoxicity or carcinogenicity of chemicals (38–40).

In the present study, we first applied our technique to evaluate whether low-dose IR could induce γ-H2AX levels and DNA damage. This evaluation was performed in AHH1 and 16HBE cells. Studies have reported a linear dose-dependent increase between the γ-H2AX values and radiation dose (0.2–6 Gy) as measured using flow cytometry and immunofluorescence detection, respectively (41, 43). A comparison of our results with data in the literature indicates that our method can sensitively detect changes in the γ-H2AX signal under exposure to γ-ray at 20 times lower doses (41). To our knowledge, this is the first report that γ-H2AX levels can be detected stably at doses as low as 0.01 Gy to observe the time effect of DNA damage using high throughput mass spectrometry quantification *in vitro*.

According to the time-effect relationship for γ-H2AX after low doses of γ-ray exposure in cells, we observed an obvious difference between the two types of cells. The γ-H2AX level in AHH1 cells increased to a greater extent than in 16HBE cells at 0.5 h post-irradiation, further suggesting that the γ-H2AX levels in AHH1 cells are more sensitive to low levels of IR exposure (41). This was supported by the fact that the γ-H2AX level in 16HBE cells quickly declined to its lowest level, approximately that observed in the non-irradiated cells after irradiation, even at a 0.2 Gy dose exposure. Moreover, we found that although H2AX is rapidly phosphorylated after treatment with low doses of γ-ray, γ-H2AX in cells first decays rapidly and then transitions to a slow decline over post-irradiation time, which is consistent with our previous report about the state of γ-H2AX induced by genotoxic compounds (38). This phenomenon further demonstrates the time dynamics of DNA damage and repair, that is, early DNA repair is fast and late repair is relatively slow.

As mankind moves into space, the space radiation environment poses considerable harm to the lives and health of astronauts (5, 6, 44). The IR hazards to astronauts inside and outside the aircraft cabin mainly depend on the composition of space radiation particles, among which protons are the most important components, followed by heavy ions (45, 46). Since AHH1 cells are more suitable for γ-H2AX research, the γ-H2AX levels in AHH1 cells after protons and heavy ions exposure were further profiled (47, 48). Our results indicated that the γ-H2AX levels under protons and heavy ions irradiation all showed a definite dose-dependent relationship, but a poor relationship appeared for heavy ions because of severe cell deterioration and even death. Higher γ-H2AX levels and more cell death induced by low-dose heavy ions irradiation at early stages could be attributed, at least in part, to the higher LET value of heavy ions. The higher the LET value of irradiation is, the more serious the degree of DNA damage caused by irradiation (49–51). In this study, the LET value of 100 MeV protons is 0.8 keV/μm and that of heavy ions is 30 keV/μm. As a consequence, the DNA damage caused by heavy ions is more intense than that caused by protons after the same dose of irradiation at early stages, resulting in higher levels of γ-H2AX. However, in the latter stage, more cells underwent apoptosis and death due to lethal damage to their DNA by heavy ions and thus the expression value of γ-H2AX decreased. It was also shown that heavy-ion irradiation led to poor or no recovery from impaired neurogenesis in mice at doses as low as 0.5 Gy (52), which supports the results in the present study.

Blood is the most commonly used sample in the clinic. The monitoring of γ-H2AX in blood cells is also most available for the evaluation of population exposure, especially for low-dose IR exposure. However, the γ-H2AX signal could not be detected in either WBCs or lymphocytes using previously published protocols (38). Accordingly, we further optimized γ-H2AX extraction and other experimental manipulations. Based on the newly developed estimation assay, we determine whether DNA damage and its profiles are caused under very low dose exposure to IR in human peripheral blood lymphocytes.

We first determined the *R*_γ/*T*_ for peripheral blood lymphocytes from 41 healthy human donors for the purpose of evaluating and confirming the background levels of γ-H2AX in fresh whole blood. We observed that the mean value of *R*_γ/*T*_ in normal human donors was 2.78 ± 0.16. There was a slight variation in the γ-H2AX level for different individuals, which may indicate that the number of γ-H2AX produced per DSB varies among individuals. We believe that this explanation is unlikely since the number of γ-H2AX correlates well-with the DSBs effects that were measured with other techniques (30, 53, 54). A more likely possibility is that an inherent difference exists in γ-H2AX levels among humans (54, 55). It has been reported that γ-H2AX levels can vary due to genetics and underlying conditions, contributing to differences in the DNA damage response and cell cycle checkpoint activation (55) and resulting in changes in the expression of γ-H2AX detected in peripheral blood. Regardless of the mechanism behind this γ-H2AX signal variation, it would be interesting to examine whether this phenomenon is linked to the sensitivity of IR exposure (56, 57) and therefore could be used, as a part of radiotherapy planning, to identify IR-sensitive individuals. In addition, γ-H2AX levels in healthy individuals which was irradiated with 4 Gy of γ-ray increased 2.8-fold at 0.5 h post-irradiation compared to those of the controls. This is consistent with our previous results where γ-H2AX levels increased 3-fold in 4 Gy-treated peripheral blood samples from healthy volunteers relative to those of the controls, as detected using flow cytometry (41).

In order to further validate whether the estimate assay could be used to measure the γ-H2AX response after low-dose exposure to γ-ray in blood, fresh whole blood from healthy subjects was irradiated with γ-ray at doses of 0.01–0.2 Gy, respectively. The results indicated that the γ-H2AX signal could be detected at exposures as low as 0.01 Gy. To our knowledge, this is the first report that γ-H2AX change levels were detected and showed the dose–response relationship at low doses exposure with a range of 0.01–0.2 Gy in human peripheral blood lymphocytes. Recently, several methods based on gene signatures have been reported for biodosimetry purposes in the low-dose range (16). For example, a biodosimetry study by the North Atlantic Treaty Organization (NATO) showed that single genes as well as gene signatures could be used to estimate low-dose IR exposures of 0.1–6.4 Gy in 2–3 ml whole blood samples (58). The γ-H2AX MS assay showed higher sensitivity levels and stability.

Freeze thaw stability was studied for whole blood and purified lymphocyte samples because it is important in the clinical setting and for investigations of population exposure (59). Fresh whole blood was stored in an ice bath for extended periods of 10 h, and purified lymphocytes were stored in a −80°C freezer for long periods of 8 months. The results suggest no significant loss of the γ-H2AX MS signal (data not shown).

## Conclusions

In conclusion, this paper demonstrates an estimation assay based on mass spectrometry quantification of γ-H2AX for low-dose exposure to IR. For the first time, a marked variation in the γ-H2AX response in the lymphocytes of human peripheral blood exposed to low-dose IR as low as 0.01 Gy was measured stably, which is more accurate than the previously reported methods by virtue of isotope-dilution mass spectrometry. This proof-of-concept study suggests the potential utility of this assay in radiation biodosimetry and environmental risk assessment for low-dose IR exposures.

## Data availability statement

The original contributions presented in the study are included in the article/Supplementary material, further inquiries can be directed to the corresponding author/s.

## Ethics statement

The studies involving human participants were reviewed and approved by Subcommittee on Human Investigation of the Beijing Institute of Radiation Medicine. The patients/participants provided their written informed consent to participate in this study. Written informed consent was obtained from the individual(s) for the publication of any potentially identifiable images or data included in this article.

## Author contributions

HG, PZ, and JX contributed to the idea and designed the experiments. HZ and MQ performed the experiments and wrote the manuscript. YL, KW, HX, MS, DX, XA, YG, and LS were involved in the data analysis. All authors have read and approved the final manuscript.

## Funding

This work was funded by Major Project (BWS18J008), the Youth Program of National Natural Science Foundation of China (31800704), and Continuous Basic Scientific Research Project (WDJC-2019-11).

## Conflict of interest

The authors declare that the research was conducted in the absence of any commercial or financial relationships that could be construed as a potential conflict of interest.

## Publisher's note

All claims expressed in this article are solely those of the authors and do not necessarily represent those of their affiliated organizations, or those of the publisher, the editors and the reviewers. Any product that may be evaluated in this article, or claim that may be made by its manufacturer, is not guaranteed or endorsed by the publisher.
