# Supplementary material for: An estimate assay for low-level exposure to ionizing radiation based on mass spectrometry quantification of γ-H2AX in human peripheral blood lymphocytes
Source: Front Public Health. 2022 Oct 28;10:1031743. doi: 10.3389/fpubh.2022.1031743 (PMC9651621; doi:10.3389/fpubh.2022.1031743)
Supplement: Supplementary file 1 [file Table_1.DOCX]

Supplementary Material

## Supplementary Tables

**Table S1** Optimization of Inhibitors for Protease and Phosphatase

| Protease and phosphatase inhibitors | *R_r/T_* (%)/Lymphocytes | *R_r/T_* (%)/WBC |
| --- | --- | --- |
| 1× and 0.0001× | n.a. | n.a. |
| 1× and 0.001× | 2.78±0.16 | n.a. |
| 1× and 0.01× | 3.12±0.19 | n.a. |
| 1× and 0.1× | 2.97±0.26 | n.a. |
| 1× and 1× | 3.09±0.31 | n.a. |

n.a. means not available.

*R_γ/T_* means ratio of γ-H2AX to total H2AX.

**Table S2** The Optimization of the Blood Volume Required

| Blood volume | 1 mL | 2 mL | 3 mL | 5 mL | 8 mL |
| --- | --- | --- | --- | --- | --- |
| *R_r/T_* (%)/Lymphocytes | n.a. | 2.83±0.14 | 2.94±0.19 | 3.10±0.12 | 3.02±0.23 |
| *R_r/T_* (%)/Leukocyte | n.a. | n.a. | n.a. | n.a. | n.a. |

n.a. means not available.

*R_γ/T_* is the ratio of γ-H2AX to total H2AX.

## Supplementary Figure


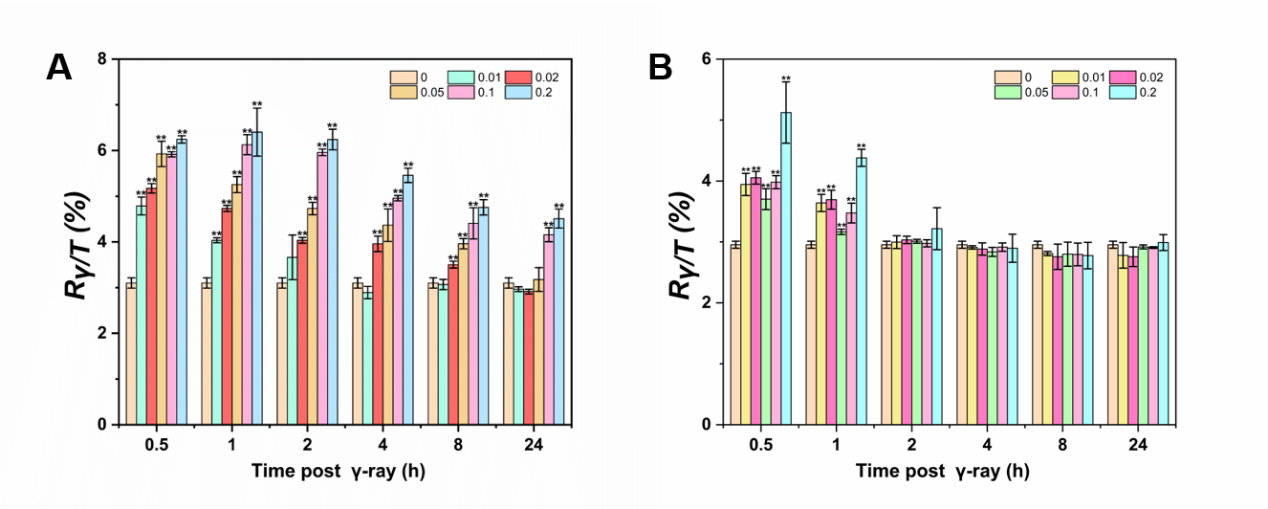


**Supplementary Figure 1.** γ-H2AX response after γ-ray exposure in AHH1 cells and 16HBE cells. (A) γ-H2AX change in 0-0.2 Gy γ-ray-irradiated AHH1 cells at various time points, (B) γ-H2AX change in 0-0.2 Gy γ-ray-irradiated 16HBE cells at various time points
